# A validated computational framework to predict outcomes in TAVI

**DOI:** 10.1038/s41598-020-66899-6

**Published:** 2020-06-18

**Authors:** Giorgia M. Bosi, Claudio Capelli, Mun Hong Cheang, Nicola Delahunty, Michael Mullen, Andrew M. Taylor, Silvia Schievano

**Affiliations:** 1 Cardiovascular Engineering Laboratory, UCL Mechanical Engineering, London, UK; 2 Centre for Cardiovascular Imaging, UCL Institute of Cardiovascular Science & Great Ormond Street Hospital for Children, London, UK; 3 Barts Health NHS Trust, University College London Hospital, London, UK; 4 UCL Institute of Cardiovascular Science, London, UK

**Keywords:** Cardiac device therapy, Interventional cardiology

## Abstract

Transcatheter aortic valve implantation (TAVI) still presents complications: paravalvular leakage (PVL) and onset of conduction abnormalities leading to permanent pacemaker implantation. Our aim was testing a validated patient-specific computational framework for prediction of TAVI outcomes and possible complications. Twenty-eight TAVI patients (14 SapienXT and 14 CoreValve) were retrospectively selected. Pre-procedural CT images were post-processed to create 3D patient-specific implantation sites. The procedures were simulated with finite element analysis. Simulations’ results were compared against post-procedural clinical fluoroscopy and echocardiography images. The computational model was in good agreement with clinical findings: the overall stent diameter difference was 2.6% and PVL was correctly identified with a post-processing algorithm in 83% of cases. Strains in the implantation site were studied to assess the risk of conduction system disturbance and were found highest in the patient who required pacemaker implantation. This study suggests that computational tool could support safe planning and broadening of TAVI.

## Introduction

Transcatheter aortic valve implantation (TAVI) is an established treatment for patients with severe aortic stenosis (AS) who are deemed unsuitable for surgery^1–5^. Since the first-in-man implantation in 2002^6^, TAVI technology has experienced a tremendous amount and pace of progress, thanks to developments in pre-procedural imaging assessment, operator experience, and engineering device research^7^, so that a treatment that was designed for high-risk patients only, is now shifting toward lower surgical risk case applications^8,9^ with many companies presenting innovative valved stent designs to overcome most of the limitations of the first-generation TAVI devices^5,7,10^.

However, TAVI still presents complications, mainly related to device sizing and positioning. If oversized, the TAVI device might cause vascular injury, such as aortic dissection, perforation or rupture of the aortic annulus;^11,12^ if undersized, it may present anchoring problems, embolization and paravalvular leakage (PVL), reported in 65–89% of cases^13,14^. Conduction abnormalities commonly (12–35%) occur following TAVI^15–18^, leading to the need of permanent pacemaker (PPM) implantation, more frequently required with self-expandable devices (28% vs. 6% of balloon-expandable choices)^19^ likely due to their material properties and larger sizes resulting in higher forces applied to the adjacent native conduction system^1,18,20^.

These complications highlight the importance of accurate interventional planning, appropriate device choice and correct patient selection. In this context, computational simulations, an already established tool to support engineering device development, could be useful, if integrated with a patient-specific approach, to support clinical decision making and introduce new technologies in clinical practice. Despite increasing acknowledgement of the usefulness of patient-specific computational modelling from regulatory agencies^21,22^, academia^23–26^ and industry^27,28^, clinical adoption of such methods is mostly limited to individual cases^29–33^.

Therefore, the aim of this work was to explore the use of a patient-specific computational modelling framework to predict outcomes of TAVI procedures in a series of 28 retrospective cases, treated with the balloon-expandable Edwards SapienXT® (Edwards Lifesciences LLC, Irvine, CA, USA) and the self-expandable CoreValve Revalving System® (Medtronic CoreValve, Medtronic Inc., Minneapolis, MN, USA). The modelling framework was based on patient-specific anatomical information and population-specific material properties to estimate the final geometrical configuration of the stents, PVL and onset of conduction abnormalities.

## Methods

### Patient population

Twenty-eight patients, who underwent successful TAVI at the Heart Hospital (London, UK) to treat severe aortic stenosis, were retrospectively included in this study. Ethical approval and need for informed consent for this study were waived by the NHS Health Research Authority: the data used in this study were collected for clinical purpose, and retrospectively reviewed and anonymised for research. All methods were performed in accordance with the relevant guidelines and regulations.

Fourteen patients (mean age at intervention = 79.3 + /−8 years, 9 males; Table 1) underwent implantation of a SapienXT (one 23 mm, nine 26 mm and four 29 mm size devices). The average volume of the calcific deposit, found from computerized tomography (CT) images analysis, was 726mm^3^. Immediate post-TAVI PVL assessed by echocardiography was present in twelve cases – nine trivial and three mild. Pre-operative heart rhythm was abnormal for six patients: S-2, S-3, S-5, S-6, S-10, S-11, but did not require treatment.

**Table 1:** SapienXT patients selected for the study.

Patient	Age at TAVI	Gender	Volume of leaflet calcium [mm^3^]	AV peak gradient [mmHg]	SapienXT size [mm]	Fluoro Diam [mm]	FE-Fluoro Diameter Difference [mm] (%)	Paravalvular leakage
S-1	85	M	385	46	29	25.3	1.0 (3.8)	trivial in NCC
S-2	77	M	627	48	29	27.1	−0.7 (−2.7)	trivial in NCC
S-3	78	M	220	67	26	26.3	−0.4 (−1.6)	trivial in NCC
S-4	59	F	1121	67	26	23.2	0.8 (3.6)	trivial in RCC
S-5	69	F	690	70	26	23.7	1.8 (7.3)	1 trivial jet in RCC-LCC, 1 trivial jet in NCC
S-6	76	M	751	70	26	25.0	−0.1 (−0.2)	No PVL
S-7	85	M	527	72	26	25.1	0.6 (2.5)	trivial in RCC, almost absent
S-8	78	M	1997	72	26	25.4	0.9 (3.3)	1 mild jet in RCC, 1 trivial jet in NCC
S-9	88	F	380	73	23	21.4	0.8 (3.5)	1 trivial jet LCC-NCC, 1 mild+ jet RCC-NCC
S-10	83	M	1478	76	29	25.5	0.5 (1.8)	trivial in NCC-RCC
S-11	78	F	221	78	26	22.7	2.7 (11.2)	trivial in RCC-LCC
S-12	83	M	553	83	26	25.6	−0.6 (−2.3)	Trivial
S-13	81	M	824	100	29	26.4	−0.1 (−0.3)	1 mild jet in RCC, 1 trivial jet in RCC-NCC
S-14	90	F	385	103	26	23.5	1.3 (5.5)	No PVL
Average + /− std	79.3 ± 8.0		726 ± 503	73 ± 16		24.7 +/− 1.6	0.6 + /− 0.9 (2.5 + /− 3.9)	

The last three columns report the stent diameter measured from fluoroscopy, the % difference of this diameter from computational results and the clinical outcome in terms of paravalvular leakage (PVL). RCC = right coronary cusp; LCC = left coronary cusp; NCC = non-coronary cusp.

Fourteen patients (mean age at intervention 81.5 + /−10.2 years, 8 males; Table 2) received a CoreValve (seven 26 mm and seven 29 mm size devices). The average volume of the calcific deposit was 686mm^3^. Immediate post-TAVI PVL assessed by echocardiography was present in twelve cases – three trivial and nine mild regurgitation. Seven Corevalve patients (C-1, C-4, C-5, C-7, C-9, C-10, C-12) had conduction abnormalities before TAVI (four had atrial fibrillation/flutter, three had first degree heart block), and two of them had a PPM already implanted (C-4 and C-7). There was no change in conduction abnormalities post-TAVI for these seven patients. One patient (C-14) who was in sinus rhythm before the percutaneous procedure, underwent implantation of a PPM due to the onset of conduction disturbances (i.e. atrio-ventricular block) after TAVI.

**Table 2:** CoreValve patients selected for the study.

Patient	Age at TAVI	Gender	Volume of leaflet calcium [mm^3^]	AV peak gradient [mmHg]	CoreValve size [mm]	Fluoro Diam [mm]	FE-Fluoro Diameter Difference [mm] (%)	Paravalvular leakage
C-1	91	F	26	14	26	21.1	0.1 (0.4)	No PVL*
C-2	64	F	957	31	26	19.7	0.6 (2.8)	1 mild jet in RCC-NCC 1 moderate jet in NCC-LCC
C-3	68	F	201	35	29	23.1	0.9 (3.9)	No PVL but free echo space
C-4	75	M	109	47	29	22.6	0.8 (3.6)	1 trivial jet in NCC 1 mild jet in LCC-NCC
C-5	78	M	431	61	29	22.2	1.6 (7.1)	1 trivial jet in NCC-LCC 1 mild jet in RCC
C-6	90	F	737	61	26	21.7	−0.4 (−2.0)	mild-moderate in LCC
C-7	74	M	1357	67	29	23.7	0.2 (0.7)	mild-moderate in RCC
C-8	68	M	1245	70	29	22.2	1.8 (7.6)	mild in NCC
C-9	81	M	922	73	29	23.6	0.1 (0.6)	trivial-mild in NCC-RCC
C-10	92	M	1098	76	26	22.0	−0.2 (−0.7)	1trivial jet in RCC 1 mild-moderate jet in LCC-NCC
C-11	91	M	596	83	26	19.2	1.4 (7.1)	trivial in LCC-NCC
C-12	87	M	1508	83	29	23.7	1.3 (5.3)	trivial in NCC-RCC
C-13	93	F	344	90	26	22.6	0.1 (0.6)	mild in NCC-LCC
C-14	89	F	53	93	26	19.2	0.3 (1.4)	trivial in RCC
Average + /− std	81.5 ± 10.2		685 ± 506	63 ± 24		21.9 +/− 1.6	0.6 + /− 0.7 (2.7 + /− 3.1)	

The last three columns report the stent diameter measured from fluoroscopy, the % difference of this diameter from computational results and the clinical outcome in terms of paravalvular leakage (PVL). RCC = right coronary cusp; LCC = left coronary cusp; NCC = non-coronary cusp. ^*^Small central leakage was detected with echocardiography.

Post-procedural fluoroscopy images were analysed to measure device diameter at the valve level at the end of stent expansion. Post-TAVI echocardiography studies and reports were reviewed by an experienced cardiologist to assess presence and location of PVL. These procedural outcomes were used for comparison with computational results^34^.

### TAVI computational analyses

Computational analyses mimicking TAVI were performed in a blinded fashion using finite element (FE) solver Abaqus 6.14/Explicit (Dassault Systèmes Simulia Corp., Providence, RI, USA) under the hypothesis of quasi-static conditions.

**Figure 1:**
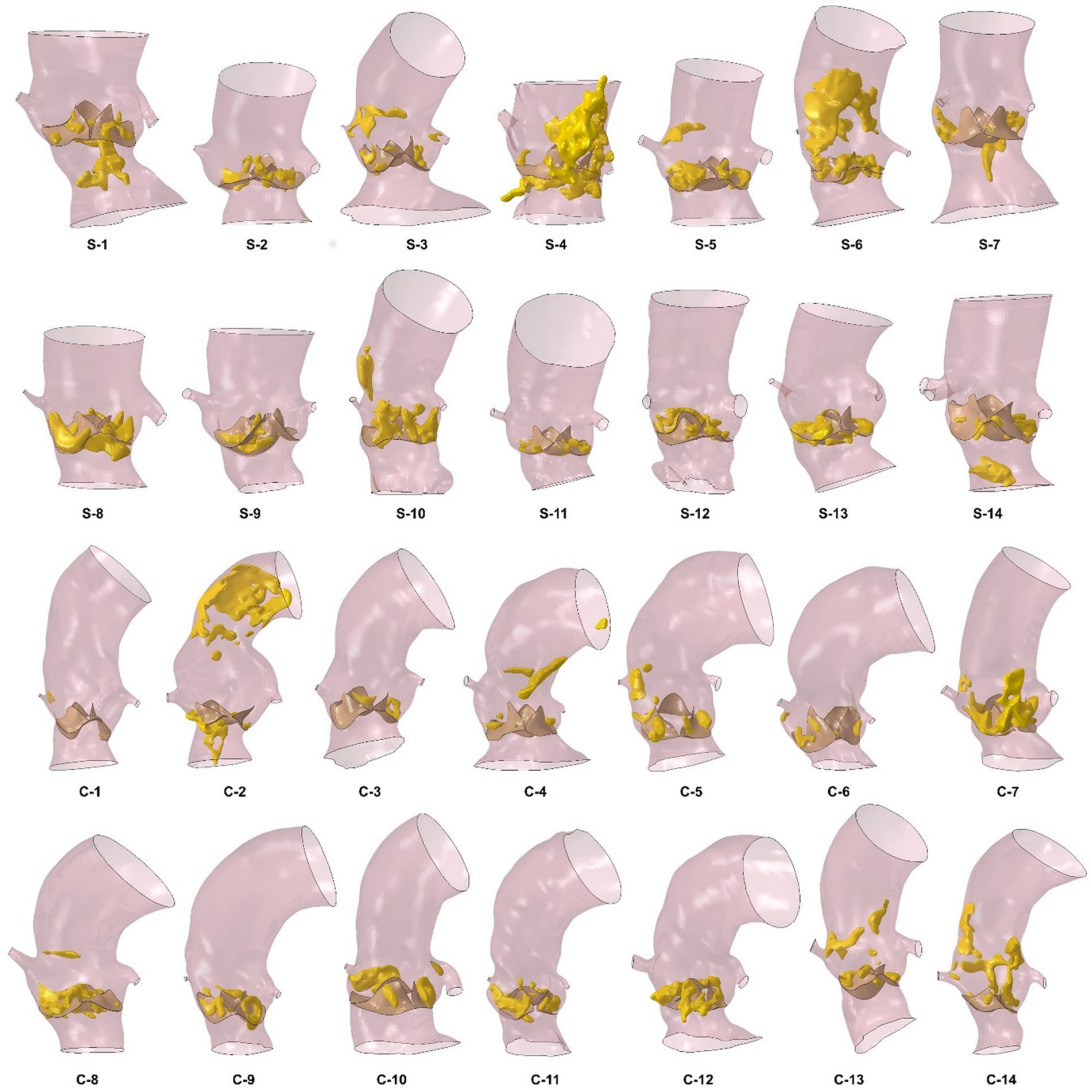
3D reconstruction for the 28 patients considered in this study; the calcific plaques are represented in yellow.

Patient-specific implantation site anatomies were created by processing pre-intervention CT images, as described in^34,35^ (Fig. 1). The 3D anatomical model of each patient implantation site included aortic root with origin of the coronary arteries, valve leaflets, ascending aorta and calcific deposits; the adopted material parameters are listed in Table 3.

**Table 3:** Material parameters.

	Young modulus [MPa]	Poisson’s ratio	Yield stress [MPa]	Density [kg/m^3^]	Thickness [mm]
Artery	7.78^49^	0.45	—	1,250^50^	1.9^49^
Leaflets	6.375^49^	0.45	—	1,250^50^	0.5^49^
Calcium	250	0.3^51^	0.25	2,000	—
MP35N	232,800	0.3	414	8,000	—
PET	600^52^	0.4	—	1,380	0.06

Balloon valvuloplasty (BAV) was simulated before TAVI using the same balloon model as that to expand the SapienXT device, non-compliant with membrane elements (Table 3). Both TAVI stent models were designed in their expanded configuration starting from micro-CT scans^30^ and meshed with beam elements. The biological valves mounted into the TAVI devices were neglected, as only the deployment phase was of interest^36^. The SapienXT stents were modelled as elastoplastic (MP35N, Table 3) whilst the material properties of the CoreValve stents, made of a shape memory alloy with superelastic behaviour at body temperature, were described by a built-in Abaqus subroutine^37^ using the same parameters as in Bosi *et al*.^30^.

In the simulations, both devices were crimped to the size of the delivery catheter by means of a cylindrical surface coaxial with the device. BAV was virtually replicated by positioning inside the patient-specific anatomy the previously deflated balloon^34,35^, subsequently inflated to a pressure of 5 atm and finally deflated again. The position of the stent in the patients’ implantation sites was chosen according to TAVI guidelines both during the actual procedure and in the blinded simulation: the SapienXT device was virtually implanted in sub-coronary position, 1/3 below the annulus of the native aortic valve^38^, whilst for the CoreValve devices the implantation depth was ≤6 mm below the native annulus plane^20^. SapienXT stents were deployed by balloon-expansion (same phases as for BAV) and CoreValve stents by release from the delivery sheath, pulling back (80 mm axial displacement) the cylindrical surface covering the catheter^39^.

### Parameters of interest

The computed stent diameter at the level of the TAVI valve was compared with the diameter measured from fluoroscopy images for all cases; before measuring the projected diameter the FE model was first oriented in the same projection as the x-rays as TAVI devices do not always deploy in a circular configuration.

Presence of potential PVL in the simulations was identified using an algorithm designed in house (Matlab, MatWorks, MA, US)^30,34^ to quantify the contact between implantation site and device along its length, and, therefore, identify gaps between artery and device. A gap between implantation site and stent was considered to be a source of PVL if it was continuous along the entire length of the SapienXT stent and in the proximal 12 mm portion of the CoreValve device, along the stent skirt. This was compared to presence and position of PVL jets measured from echocardiography images of each case, with location described by the three aortic valve sinuses.

Max Principal Strains on the aortic root portion below the coronary ostia, in particular in the non-coronary cusp (NCC)- right coronary cusp (RCC) area where the left bundle branch is located, were assessed in the FE model: high values imply higher forces exerted by the proximal portion of the expanded TAVI device to stretch the implantation site, and therefore potential risk of conduction abnormalities^40^.

## Results

**Figure 2:**
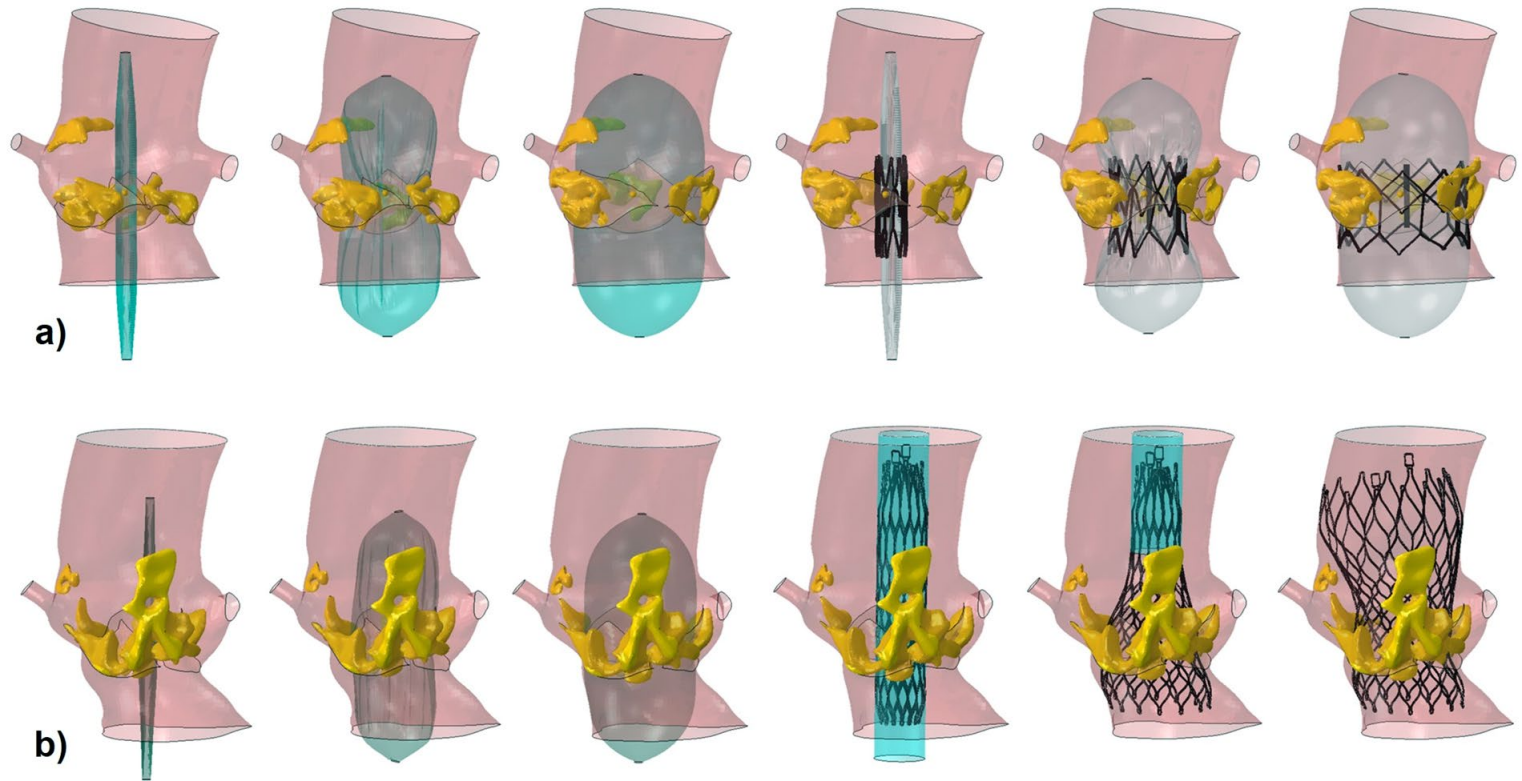
Simulation phases of BAV followed by (**a**) SapienXT 26 mm implantation in patient S-5, and (**b**) CoreValve 29 mm implantation in patient C-7.

Figure 2(a,b) shows two examples of the simulation phases of BAV followed by SapienXT and CoreValve implantation respectively.

**Figure 3:**
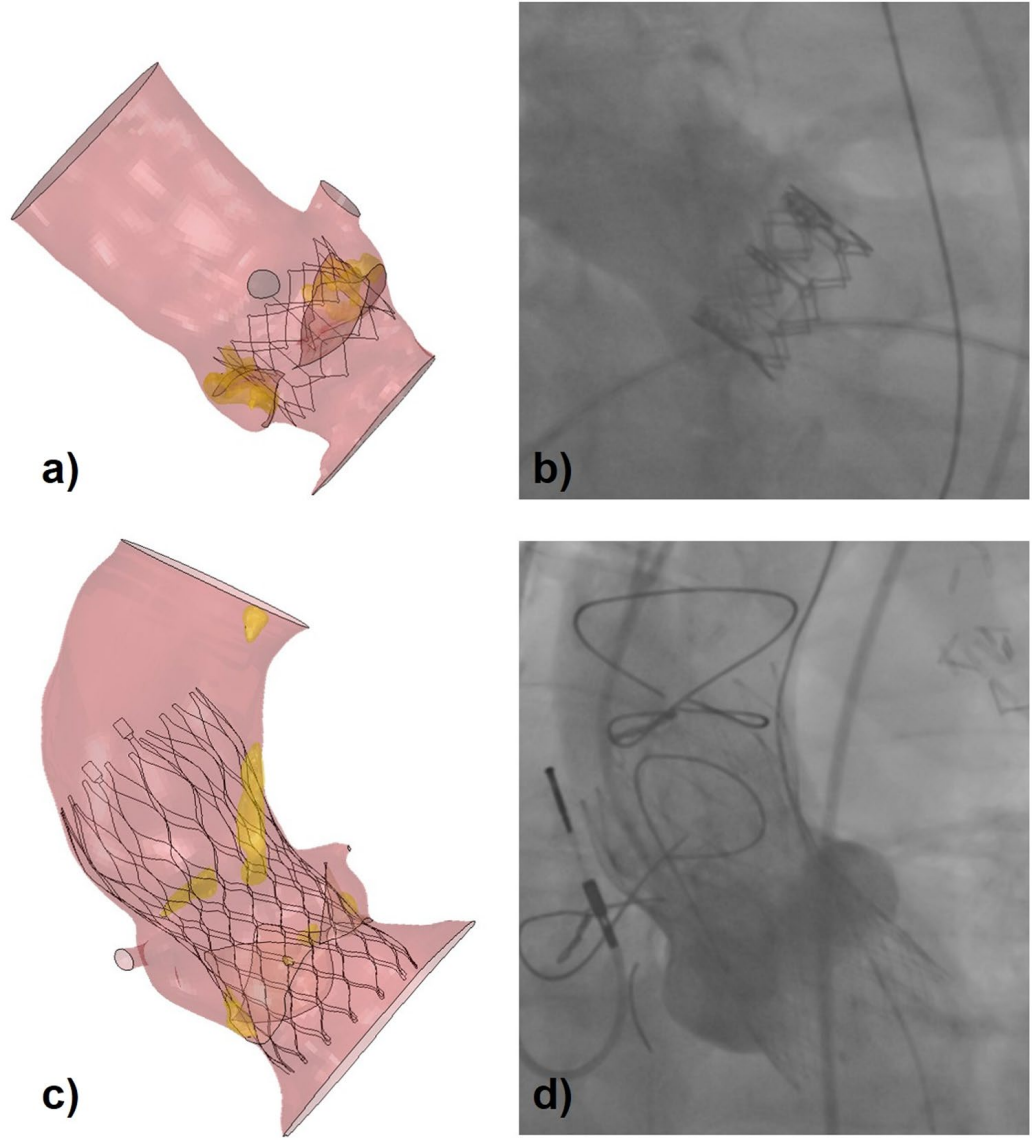
(**a**,**c**) Final configuration of the virtually implanted SapienXT 26 mm in patient S-12 and of CoreValve 29 mm device in patient C-4 respectively, compared with (**b**,**d**) fluoroscopy images acquired during the real procedure for the same patients.

Good agreement was found between FE simulations and fluoroscopy image in terms of diameters reached at the end of device deployment for both stents: in the SapienXT cases, the mean difference was 0.6 + /−0.9 mm (2.5 ± 3.9%), with a maximum under-expansion error in patient S-2 of -0.7 mm (-2.7%) and a maximum over-expansion error of 2.7 mm (11.2%) in patient S-11 (Table 1). In the CoreValve cases, the average difference was 0.6 + /−0.7 mm (2.7 ± 3.1%), with a maximum under-expansion error in patient C-6 of -0.4 mm (-2.0%) and a maximum over-expansion error of 1.8 mm (7.6%) in patient C-8 (Table 2). In Fig. 3, two examples of FE results are reported for comparison against the corresponding fluoroscopy images.

**Figure 4:**
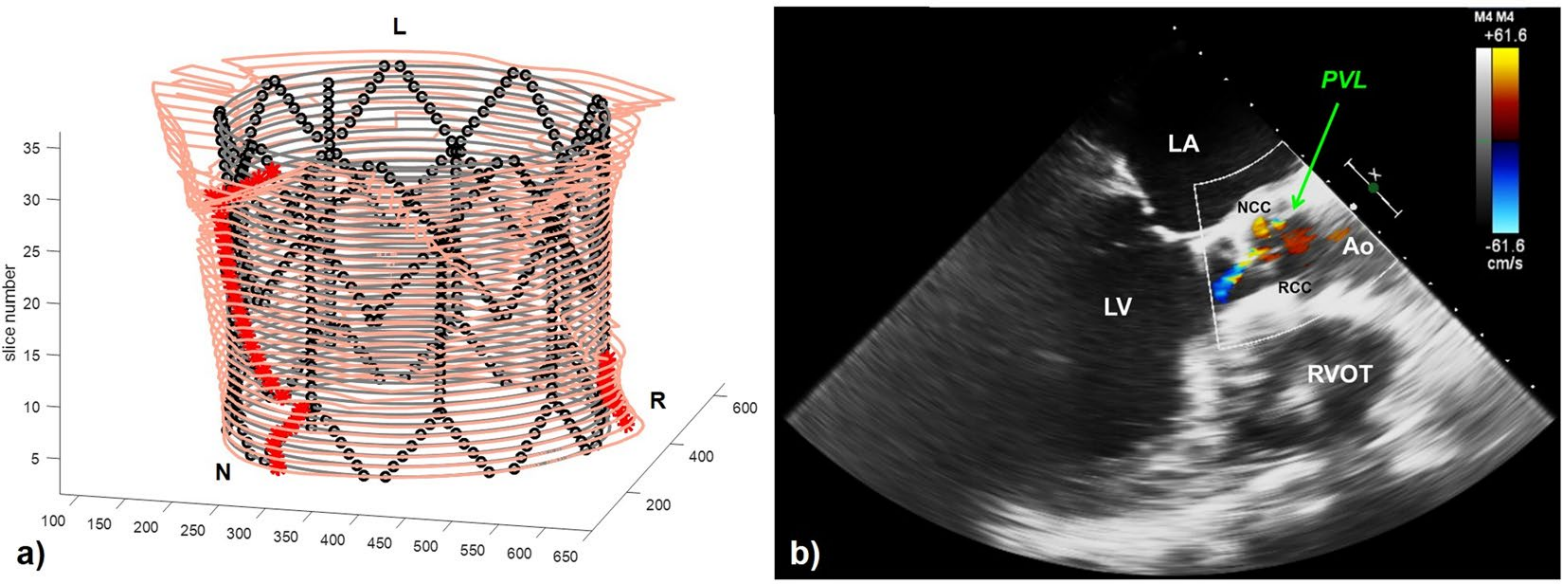
(**a**) Matlab elaboration of the FE results for patient S-3, highlighting the presence of PVL in the non-coronary cusps (NCC) of the valve (red asterisks). (**b**) Mid-oesophageal long axis view for the same patient: a trivial jet of paravalvular regurgitation is showed in the same position. The green arrow points at the PVL jet. LV = left ventricle; LA = left atrium; RVOT = right ventricle outflow tract; Ao = Aortic root; R = right coronary cusp; N = non-coronary cusp.

**Figure 5:**
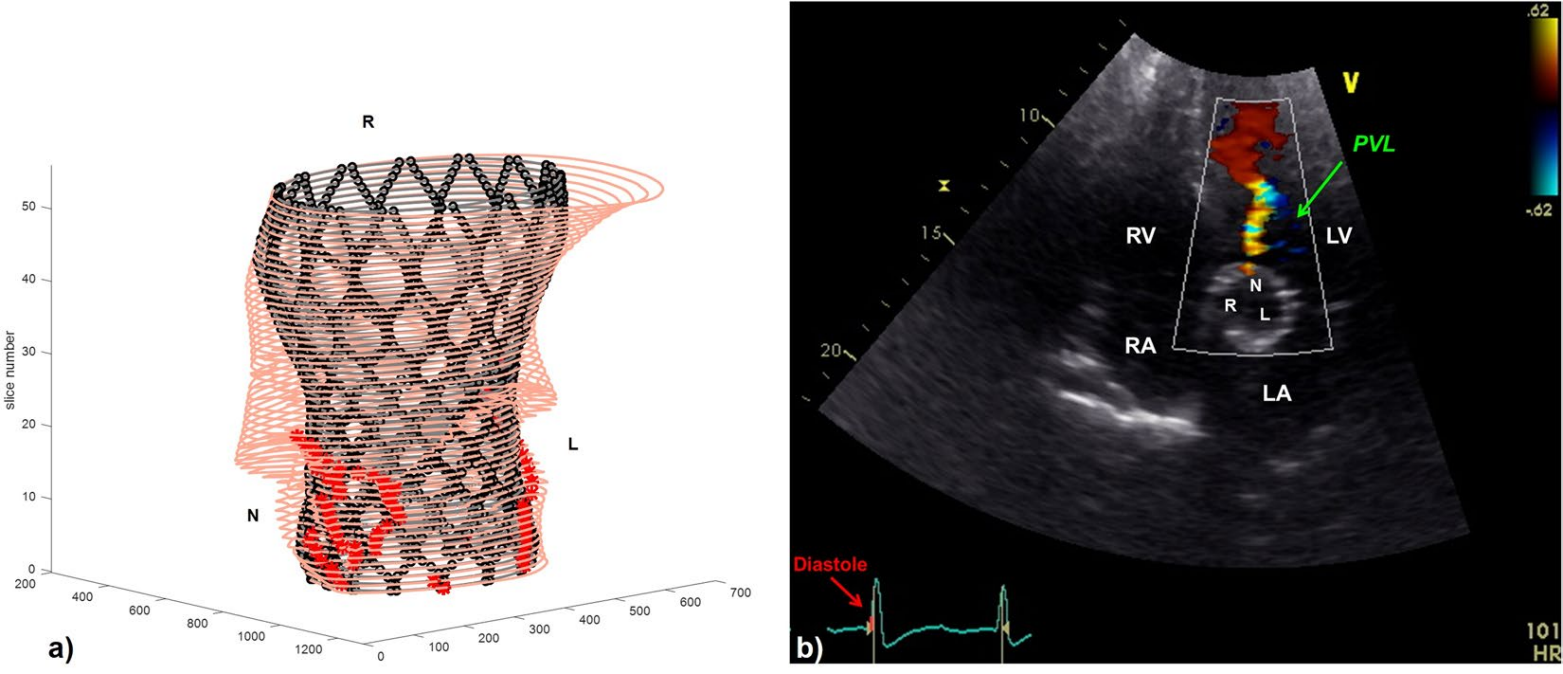
(**a**) Matlab elaboration of the FE results for patient C-8, highlighting the presence of PVL in the non-coronary cusps (NCC) of the valve (red asterisks). (**b**) Transthoracic echocardiographic 5 chamber view for the same patient: one jet of paravalvular regurgitation (PVL) is showed by the green arrow in the non-coronary cusp. The ECG shows that the image was taken during diastole, i.e during the bioprosthetic valve closure. RV = right ventricle, RA = right atrium; LA = left atrium; LV = left ventricle; R = right coronary cusp; L = left coronary cusp; N = non-coronary cusp.

The presence/lack of PVL was correctly identified by the computational model in 79% of SapienXT cases and in 86% of CoreValve cases. All patients who did not present any PVL were correctly detected by the code, thus showing good specificity of the computational framework. Among the patients correctly identified with PVL, the exact location was recognised in 67% of SapienXT and 45% of CoreValve cases. Figures 4 and 5 report two examples of PVL analysis. Figure 4 shows the trans-oesophageal echocardiography for SapienXT patient S-3, highlighting one trivial jets of PVL at the NCC and the corresponding Matlab graph showing two channels at the NCC and RCC, only the first being continuous along the entire length of the stent and therefore indicating PVL. Figure 5 shows post-TAVI echocardiography for CoreValve patient C-8, with one trivial jet of PVL at the NCC, and the corresponding computational graph with a partial contact gap, identified by the red asterisks, in the same position; this graph shows also another possible leak in the LCC, which however was not detected in the clinical assessment.

**Figure 6:**
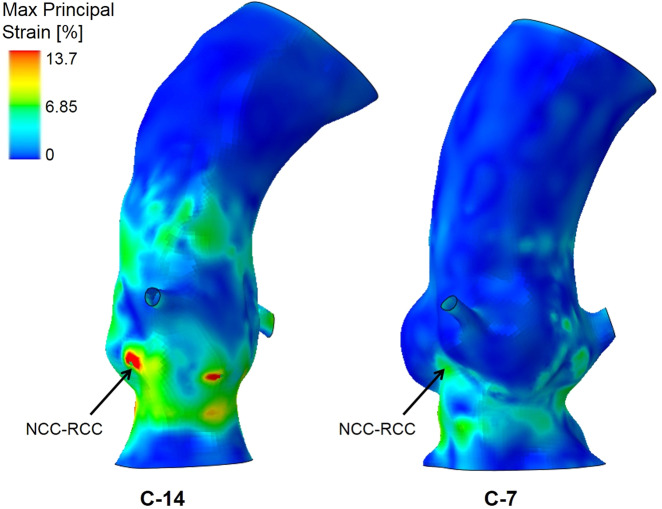
Colour map of Max Principal Strains for patient C-14 and C-7 calculated by the computational model. The highest average value in the region below the coronary arteries was found for patient C-14, whilst for example patient C-7 presents strains close to the average value for the cohort. NCC = non-coronary cusp; RCC = right coronary cusp.

In the CoreValve cohort, the average Max Principal Strain in the region below the coronary ostia (Table 4) was found to be highest in patient C-14 (4.7%), almost double if compared to the average value for all the patients (2.5 ± 1.1%) in that same region. The maximum value of the Max Principal Strain was 24.5% for the same patient (C-14), between the NCC and the RCC. Figure 6 shows the strain colour map for this patient in comparison with patient C-7, who presented Max Principal Strain values close to the average. Patient C-14 was at highest risk of PPM implantation according to the computational results and it was the only case in this cohort of patients, in which a PPM was clinically required.

**Table 4:** Max Principal Strains on the implantation site for the cohort of 14 patients who underwent the implantation of CoreValve device.

Max Principal Strains in the implantation site [%]			
Patient	Average value	Absolute max	Max in NCC-RCC
C-1	4.5	12.9	15.7
C-2	2.1	9.7	9.7
C-3	3.2	9.9	14.8
C-4	2.1	22.9	22.9
C-5	1.5	5.3	9.9
C-6	1.2	8.4	8.4
C-7	2.3	9.4	12.7
C-8	1.4	4.6	5.1
C-9	1.7	4.9	10.8
C-10	2.6	14.0	14.0
C-11	2.5	15.4	15.4
C-12	2.2	14.0	14.0
C-13	2.9	15.3	15.3
C-14	4.7	24.5	24.5
Average + /− std	2.5 ± 1.1	12.2 ± 5.2	13.8 ± 5.2

In the first column, the average value found in all the areas of the implantation site below the coronary ostia; in the second column, the absolute maximum value on the entire implantation site; in the last column, the maximum stress in the region between the right and non-coronary leaflets, where the left bundle branch is located.

## Discussion

In this work, patient-specific computational models were implemented for 28 TAVI cases (14 SapienXT and 14 CoreValve) using a previously validated framework for modelling the TAVI population implantation site^34^. The main aim of this study was to test the capability of the computational method to predict outcome parameters of clinical interest. The FE framework captured well the TAVI stent diameter at the end of the implantation procedure and the presence/lack of PVL, one of the most common clinical complication post-TAVI. In addition, FE results could be used to provide information on each patient risk for PPM after TAVI with CoreValve.

The comparison between the implanted stent diameters from the presented computational modelling framework with clinical fluoroscopy measurements showed a small mean difference (<3%) across the entire cohort of patients, thus confirming that the FE model is well set up in terms of geometrical and mechanical conditions to capture realistically the expansion of the devices in a wide range of patient-specific implantation sites.

Clinical assessment of post-TAVI PVL presence, location and severity, evaluated with echo color Doppler measurements^41^ is technically challenging and highly inter-operator dependent, since different cross-sectional views of the device might identify different PVL, in terms of both severity and position. In the FE simulations, PVL risk was assessed based on a purely geometrical analysis, without fluid-dynamics information. The post-processing Matlab code allowed automatic quantification of the interaction between the deformed implantation site and the implanted TAVI stent, and of the potential suboptimal apposition that would be source of PVL. Considering the entire patient cohort, the mathematical model was able to identify the presence/lack of PVL in 83% of cases, thus demonstrating good sensitivity. In terms of location, the code could identify the position of the PVL jets in half of the cases, with higher success rate for the SapienXT cohort compared to the CoreValve cases. This could be explained by the following factors:

1. different echocardiography cross-sectional views of the device might detect different PVL locations, especially in the case of a longer device such as the CoreValve;
2. different time frames selected for the analysis of the PVL jet position during the diastolic phase may show different locations, thus adding this uncertainty to the normal inter-operator evaluation;
3. the orientation of the TAVI leaflets in relation to the native valve leaflets might have an influence in the regurgitant flow position; the TAVI leaflets were neglected in this study and therefore this aspect could not be evaluated.

Refinements of the algorithm would improve the identification of PVL location and to detect the severity of the leak by extrapolating gap volumes and geometrical complexity.

The strain distribution on the implantation site was analysed to study the effect of TAVI on the conduction system. The Max Principal Strains parameter resulting from FE analysis, assessed below the coronary ostia both in terms of average and max value, appeared to be a good predictor for onset of conduction abnormalities leading to PPM implantation in this patient population; the parameter was the highest for the patient who underwent PPM, thus providing a potential new monitoring parameter. Importantly, this patient did not present any irregular heart rhythm pre-TAVI, hence reinforcing our hypothesis. A study with a larger cohort of TAVI patients who underwent PPM implantation is necessary to further test this hypothesis and to define strain thresholds which might be indicative of the need of PPM implantation. A recent numerical study focused on contact pressure and the implantation site regions of contact pressure – parameters also related to strains – and found these to be associated with the occurrence of new conduction abnormalities^42^.

Recently, multi-modality imaging, i.e. conventional CT combined with cardiac magnetic resonance, has demonstrated useful for pre^43^ and post-TAVI evaluation, especially to quantify residual aortic regurgitation^44^.

Alongside conventional patient assessment, access to the personalised computational framework here proposed might play an important role for predicting and quantifying potential outcomes when different treatment options are available, in borderline cases, thus adding further useful information to the clinical decision-making process. Importantly, such an engineering tool could be integrated regularly in clinical practice as based purely on routine clinical assessment data, without requiring additional patient information; the current time-scale for an expert technician to obtain computational results, starting from a dataset of routinely acquired CT images (average quality), is estimated at 6 hours (2 hours for CT image analysis, post-processing and 3D anatomy meshing; 1 hour for setting up the FE analysis; and 3 hours for the simulation to run on an average workstation, and to analyse the results). Therefore, with the current methodology, outcomes could be quantified and provided to the clinical care team in a day. The duration of this process will be improved and optimized, by increasing computational resources and introducing automatization in some of the steps.

Recently, thanks to the promising preliminary results of TAVI in intermediate-risk patients^9^, a paradigm shift toward the selection of lower surgical risk cases for the percutaneous procedures is taking place^8^. However, extending TAVI to this kind of patient poses new challenges, both for clinicians and device manufacturers; younger patients, with less severe aortic valve stenosis and calcification may present procedural (safe delivery, anchoring, wall rupture) and device related (durability) hurdles^45^. In this context, increasingly refined patient-specific computational models can potentially increase the safety of such patients, by providing additional, predictive information about responses to cardiovascular device implantation in individual cases^46,47^, evaluation of different treatments options and prediction of interventions’ complications^48^.

From a different perspective, the validated FE framework and the library of retrospective patients already treated with current devices could be useful for the medical device Industry or the Regulatory Agencies to test new device designs at low costs, with fast response times and under realistic implantation site conditions. Therefore, these tools could provide a platform to improve design features and characteristics, quantifying advantages and disadvantages in a safe, realistic environment to reduce the number of animal experiments and to reduce the time to human application.

Patient-specific computational tools to date have not become an integral component of the clinical decision-making pipeline or of the product development process. With this work, we have tested the validated^34^ patient-specific computational framework in a small cohort, showing the potential and feasibility of integrating engineering methodologies in clinical practice. Larger prospective clinical studies will be necessary to fully validate these models and promote their use in clinical practice.

In conclusion, this work presented the ability of the developed patient-specific computational framework to identify clinical outcomes on a small population of cases, showing the potential to be applied in clinics and in device design. In fact, this model could be employable by medical professionals to aid the clinical decision-making process, serving as a pre-operative planning tool, helping clinicians in selecting size, type and position of the TAVI device for each patient, especially those at lower surgical risk, in order to minimize complications, but also to enhance patient safety in the early introduction of new technologies. Moreover, this framework could be used to aid the design of new devices and virtually test them in realistic human implantation sites, before animal testing, thus reducing both workflow costs and animal sacrifices. In other words, this computational tool could serve to support safe planning and broadening of minimally-invasive heart valve replacement techniques.
